# The Mechanism Underlying the ncRNA Dysregulation Pattern in Hepatocellular Carcinoma and Its Tumor Microenvironment

**DOI:** 10.3389/fimmu.2022.847728

**Published:** 2022-02-23

**Authors:** Chen Xue, Xinyu Gu, Zhengyi Bao, Yuanshuai Su, Juan Lu, Lanjuan Li

**Affiliations:** State Key Laboratory for Diagnosis and Treatment of Infectious Diseases, National Clinical Research Center for Infectious Diseases, Collaborative Innovation Center for Diagnosis and Treatment of Infectious Diseases, The First Affiliated Hospital, College of Medicine, Zhejiang University, Hangzhou, China

**Keywords:** HCC, ncRNA, epigenetic modification, functional mechanisms, TME

## Abstract

HCC is one of the most common malignant tumors and has an extremely poor prognosis. Accumulating studies have shown that noncoding RNA (ncRNA) plays an important role in hepatocellular carcinoma (HCC) development. However, the details of the related mechanisms remain unclear. The heterogeneity of the tumor microenvironment (TME) calls for ample research with deep molecular characterization, with the hope of developing novel biomarkers to improve prognosis, diagnosis and treatment. ncRNAs, particularly microRNAs (miRNAs), long noncoding RNAs (lncRNAs), and circular RNAs (circRNAs), have been found to be correlated with HCC neogenesis and progression. In this review, we summarized the aberrant epigenetic and genetic alterations caused by dysregulated ncRNAs and the functional mechanism of classical ncRNAs in the regulation of gene expression. In addition, we focused on the role of ncRNAs in the TME in the regulation of tumor cell proliferation, invasion, migration, immune cell infiltration and functional activation. This may provide a foundation for the development of promising potential prognostic/predictive biomarkers and novel therapies for HCC patients.

## Introduction

Protein-coding genes have been well studied, but protein-coding regions account for only 1.5% of the whole human genome (1). Thousands of noncoding RNA (ncRNA) sequences that are not translated into proteins were considered “junk” of transcriptional products in the past decade. However, with advancements in high-throughput RNA sequencing technologies, increasing data have revealed that ncRNA sequences play important roles in regulating various cellular processes, including signaling pathways, transcription, posttranscriptional modifications, and chromatin remodeling (2). Multitudes of ncRNA species have been discovered, and these include microRNAs (miRNAs), long noncoding RNAs (lncRNAs), and circular RNAs (circRNAs) (3–5).

With advancement in research, ncRNAs were found to exert biological functions at the RNA level and were identified as vital regulators in several physiological and pathological processes, especially in cancer (6–9). The regulatory function and underlying molecular mechanisms of ncRNAs in hepatocellular carcinoma (HCC) have also been explored. In recent five years, emerging role of lnRNAs in HCC has been well reviewed (10–12). Many ncRNAs have been identified as important drivers or inhibitors of HCC progression (13, 14). For instance, miRNA-15a-3p is downregulated in HCC tissues, and a low level of this miRNA is positively correlated with distant metastasis and poor prognosis (15). LncRNA MCM3AP-AS1 is highly expressed in HCC and associated with large tumor size, late stage and shorter survival in HCC patients (16). The expression of circTRIM33-12 is lower in HCC tissues and cell lines than in normal controls, and the level of circTRIM33-12 might be an independent risk factor for the overall survival of patients with HCC (17). All these studies predicted that ncRNAs play important roles in the carcinogenesis and progression of HCC and that specifically expressed ncRNAs might be developed as promising biomarkers for the prognosis and treatment strategy of cancer patients. While it was less reviewed that the genetic and epigenetic dysregulation lead to abnormal expression and dysfunction of various ncRNAs. Notably, ncRNAs have been discovered to participate in HCC cell malignant phenotypes (such as proliferation, migration, invasion, drug resistance), as well as immune cell development and function in the tumor microenvironment (TME).

In this review, we summarized the aberrant epigenetic and genetic alterations that cause aberrant ncRNA expression. We also discussed the functional mechanism of classical ncRNAs in the regulation of gene expression and provide examples of the far-reaching influence that these molecules have in affecting HCC processes. Furthermore, we summarized the effect of ncRNAs on the TME and laid the foundation for their applications in targeted therapy. In particular, we emphasized the role of ncRNAs in regulating tumor-associated macrophages (TAMs) in human HCC. Finally, we discussed the potential of ncRNAs as prognostic and diagnostic biomarkers and therapeutic targets in the future.

## Abnormal ncRNA Expression in HCC

**Figure 1:**
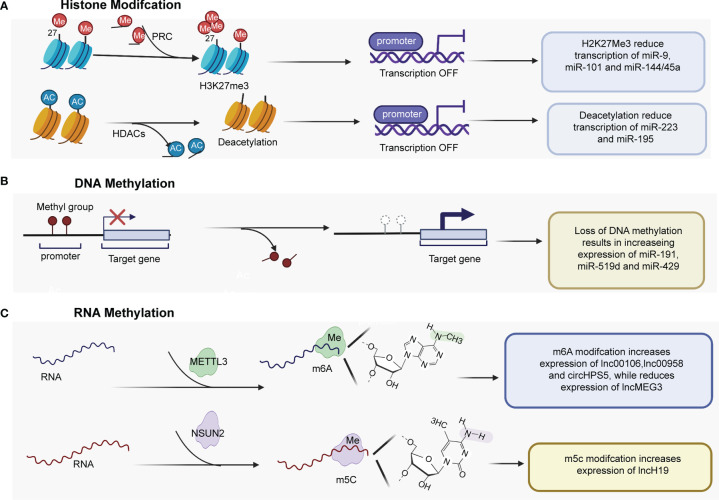
Epigenetic mechanisms play important roles in regulating the expression of ncRNAs. **(A)** H3K27me3 mediated by PRC2 regulates miRNA expression by regulating the promoters of miR-9, miR-101, and miR-144/45a. HDACs are recruited to the miR-223 and miR-195 promoter, and histone deacetylation contributes to genes downregulation. **(B)** Several tumor-promoting miRNAs have been found to be upregulated by promoter DNA hypomethylation. **(C)** m6A methyltransferase METTL3 catalyzes m6A modification of some lncRNAs and circRNA at the posttranscriptional level, promoting the expression of lnc00106, lnc00958 and cirHPS5, while reducing lncMEG3 expression level. PRC2, Polycomb repressive complex 2; HDACs, histone deacetylases; miR, microRNA; H3K27me3, trimethylation of histone H3 at lysine 27; METTL3, Methyltransferase-like 3.

As documented in the previous section, various ncRNAs were aberrantly expressed in HCC. Aberrant expression of ncRNAs can arise through many different mechanisms. The section reviews mainly focused on aberrant epigenetic mechanisms (**Figure 1**) and genetic alterations.

### Copy Number Variations (CNVs)

CNVs are the most common DNA variations in cancer cells and are particularly common in noncoding protein regions. The whole-genome sequencing data of 49 Chinese HCC patients showed that lncRNAs were amplified in HCC tumor tissues mainly located on chromosomes 1q, 8q, 17q, and 20q, while lncRNAs deleted in the tumor tissues were mostly located on chromosomes 4q, 9q, 13q, and 16q. TaqMan copy number assay of HCC tumors and normal liver tissues from 238 patients further confirmed that lncRNAs with copy number gain in >50% of HCC samples were all upregulated in tumor tissues (18). In another study, to explore the association between HCC metastasis and lncRNAs, Yang et al. (19) identified 917 recurrently deregulated lncRNAs, and 147 of these recurrently deregulated lncRNAs were found in deleted regions. For instance, the CNV-driven lncRNA FENDRR had a pattern of decreased expression (19). Zhang et al. (20) reported that lncRNA TSLNC8 is a tumor suppressor that is located on chromosome 8p12 and is frequently deleted in HCC tissues.

### Histone Modification

The dysregulation of ncRNAs is commonly mediated by various types of histone modifications, such as histone acetylation, histone deacetylation, H3K27ac, and histone H3 lysine 27 trimethylation (H3K27me3) (**Table 1**). Enhancer of zeste homolog 2 (EZH2), an essential enzymatic unit of polycomb repressive complex 2 (PRC2), is the sole histone methyltransferase that promotes H3K27me3, leading to silencing genes by regulating miRNA promoters. Many studies have reported that PRC2 mediates the downregulation of tumor suppressive miRNAs, including miR-101-1 (21, 22), miR-9 (23), and miR144/451a (24), by interacting with EZH2 in HCC (**Figure 1A**). In addition, Zhao et al. (31) found that histone writers EP300 and WDR5 bind to the circSOD2 promoter and mediate its promoter H3K27ac and H3K4me3 modification, thereby enhancing circSOD2 expression. Additionally, several studies have reported that histone deacetylation and histone acetylation play critical roles in chromatin remodeling and ncRNA regulation (25–30, 32). For example, Dong et al. (32) reported that miR-223 was downregulated in HCC tissues and cell lines, that histone deacetylase (HDAC) 9 and HDAC10 were recruited to the miR-223 promoter and that deacetylation contributed to miR-223 downregulation. In addition, deacetylation was also found to regulate the expression of miR-195 (27) (**Figure 1A**). miR-122 is an important tumor suppressor and plays an essential role in the maintenance of liver homeostasis, and the expression of miR-122 is reduced in hepatic and circulating HCC patients (33–35). DNA methylation inhibitor- and HDAC inhibitor-treated HCC cells (HepG2 and Huh7) showed upregulated pri-miR-122 levels through liver-enriched transcription factors to bind the promoter region of miR-122 (30).

**Table 1:** Oncogenic or tumor suppressive ncRNAs is regulated by histone modification in HCC.

miRNA	Expression	Role of miRNA	Regulators	The type of histone modification	References
miR-101	Downregulated	Tumor suppressor	PRC2	H3K27me3	(21, 22)
miR-9	Downregulated	Tumor suppressor	PRC2	H3K27me3	(23)
miR-144/451a	Downregulated	Tumor suppressor	PRC2	H3K27me3	(24)
miR-223	Downregulated	Tumor suppressor	HDAC9 and HDAC10	Histone deacetylation	(25)
miR-224	Upregulated	Tumor promotor	HDAC	Histone deacetylation	(26)
miR-195	Downregulated	Tumor suppressor	HDAC3	Histone deacetylation	(27)
miR-30a-5p	/	/	/	Histone acetylation	(28)
miR-17-92 cluster	Upregulated	Tumor promotor	HDAC inhibitor	Histone acetylation	(29)
miR-122	Downregulated	/	SUV39H1	Histone H3K9 methylation and histone acetylation	(30)
circSOD2	Upregulated	Tumor promotor	EP300 and WDR5	H3K27ac and H3K4me3	(31)
lncRNA MIAT	Upregulated	Tumor promotor	/	histone acetylation	(32)

### DNA Methylation

DNA methylation (including DNA hypomethylation and/or promoter gene CpG hypermethylation), an epigenetic modification, is a common factor that regulates the expression of ncRNAs in HCC (**Table 2**). DNA methylation of CpG islands within the promoter regions of tumor suppressor genes is known to suppress transcriptional initiation and thereby silence these genes. Among the different types of ncRNAs, miRNAs were the first discovered and have been the most extensively studied. Many miRNAs with tumor suppressor characteristics have been found to be silenced by promoter DNA hypermethylation (39, 40, 42, 43, 45–47). Conversely, a few tumor-promoting miRNAs have been found to be upregulated by promoter DNA hypomethylation (**Figure 1B**). For instance, Datta et al. (36) found that the CpG island of miR-1 was methylated in HCC cells (HepG2 and Hep3B cells) and tissues. miR-1 expression was lower in HCC cell tissues than in normal liver tissues. According to microRNA microarray analysis, the DNA hypomethylating agent 5-azacytidine restored the expression of miR-1 in HCC cells. He et al. (37) found that hsa-miR-191 was upregulated in HCC tissues and that hypomethylation of the CpG island of miR-191 led to high expression of miR-191 and enhanced epithelial-mesenchymal transition (EMT) in HCC. In addition, hypomethylation of the CpG island of miR-519d and miR-429 resulted in increasing expression of miR519d (38) and miR-429 in HCC (41, 44)

**Table 2:** The expression, function, and DNA methylation status of ncRNAs in HCC.

Detectable location	Methylation status	LncRNA/miRNA	Role	Expression	Targets	Function	References
HCC cells and tissues	Promoter gene CpG hypermethylation	miR-1	Tumor suppressor	Downregulated	FoxP1, MET, HDAC4	Inhibits cell cycle progression and promotes apoptosis	(36)
Tissues	Promoter gene CpG hypomethylation	miR-191	Tumor promoter	Upregulated	/	EMT	(37)
Tissues	Hypomethylation	miR-519d	Tumor promoter	Upregulated	CDKN1A/p21	Promotes cell proliferation, invasion and impairs apoptosis	(38)
Tissues	CpG island hypermethylation	miR-335	Tumor suppressor	Downregulated	/	/	(39)
Serum and tissues	Hypermethylation of the promoter	miR-132	Tumor suppressor	Downregulated	Akt pathway	/	(40)
Tissues	Hypomethylated	miR-429	Tumor promoter	Upregulated	RBBP4	/	(41)
Cell lines	Hypermethylation of CpG island	miR-148a	Tumor suppressor	Downregulated	DNMT1	Inhibits HCC cell proliferation and cell cycle progression	(42)
Tissues and cell lines	Hypermethylation of CpG island	miR-941	Tumor suppressor	Downregulated	KDM6B	Suppresses cell proliferation, migration, and invasion	(43)
Tissues	Hypomethylatedstatus	miR-429	Tumor promoter	Upregulated	PTEN	Enhances HCC migration and invasion, and EMT	(44)
Tissues	Hypermethylation of CpG island	miR-615-5p	Tumor suppressor	Downregulated	RAB24	Inhibits cell motility and metastasis	(45)
Tissues and cell lines	Hypermethylation	miR-142	Tumor suppressor	Downregulated	TGF-β	Suppresses cell vitality, proliferation, EMT and angiogenesis	(46)
/	Hypermethylated	miR-148a	Tumor suppressor	/	NF-κB	/	(47)
Serum and tissues	Promoter gene CpG hypermethylation	lnc SRHC	Downregulated	Downregulated	/	Inhibits cancer proliferation	(48)

In recent years, the intriguing features of lncRNAs and circRNAs and DNA methylation have also been explored. For example, lncRNA SRHC was found to be downregulated in HCC tissues and serum samples compared with normal liver tissues and matched serum samples (48). The downregulation of SRHC was induced by DNA methylation of CpG islands in the promoter region of SRHC (48). In addition, Xu et al. (49) reported a list of dysregulated circRNAs (including circAHSA2P, circDCUN1D4, circCCNL2, and circCLEC16A, etc.) which were correlated with DNA methylation alterations based on genome-wide DNA methylation and RNA sequencing analysis of 20 pairs of HCC tissues and normal liver tissues.

### RNA Methylation

Recently, RNA methylation of ncRNAs has gained much attention (50–54). For instance, M6A, a well-known type of RNA modification at the posttranscriptional level, has been reported to be involved in the expression of miRNAs, circRNAs and lncRNAs in HCC (**Table 3** and **Figure 1C**). For example, Liang et al. (55) reported that the expression of lncRNA 00106 was higher in HCC tissues than in normal liver tissues, and the results of m6A RNA immunoprecipitation (RIP) and qRT–PCR assays showed that m6A was significantly enriched in HCC cells compared with THLE-2 cells. Further gain- and loss-of-function experiments revealed that methyltransferase-like 3 (Mettl3), an m6A methyltransferase, mediated lncRNA 00106 stability in HCC, promoting lncRNA 00106 expression by facilitating m6A modification (55). Similarly, the expression of lncRNA 00958, lncRNA MEG3, and circRNA HPS5 was also found to be regulated by m6A modification in HCC (56–58). Moreover, lncRNA H19 was abnormally increased and had a tumor-promoting effect in HCC. Mechanistically, lncRNA H19 was found to be a specific target for NSUN2, which is an RNA methyltransferase responsible for m5C modification (59).

**Table 3:** The expression, RNA methylation regulators and the type of RNA methylation of lncRNAs/circRNAs in HCC.

Regulators	lncRNA/circRNA	Expression	Role	The type of RNA methylation	References
METTL3	lnc00106	Upregulated	Tumor promotor	m6A methylation	(55)
METTL3	lnc00958	Upregulated	Tumor promotor	m6A methylation	(56)
METTL3	lncMEG3	Downregulated	Tumor suppressor	m6A methylation	(57)
METTL3	circHPS5	Upregulated	Tumor promotor	m6A methylation	(58)
NSUN2	lncH19	Upregulated	Tumor promotor	m5C modification	(59)

## Functional Mechanisms of ncRNAs in HCC

Although ncRNAs do not encode proteins, they regulate gene expression levels at various levels (epigenetic regulation, transcriptional regulation and posttranscriptional regulation, etc.) in the form of RNA. The functional mechanism of ncRNAs is complex and is not yet fully understood. Previously published studies mainly focused on the following mechanism of gene expression regulation.

### Functional Mechanisms of lncRNAs in HCC

#### lncRNA-Mediated Epigenetic Modification

One of the important functional mechanisms by which lncRNAs regulate gene transcription is *via* the binding and recruitment of epigenetic modifiers to specific genomic regions in HCC (60), inducing aberrant epigenetic modifications such as DNA demethylation and hypermethylation. For instance, lncRNA DDX11-AS1 directly binds with the active histone methyltransferase EZH2, which regulates H3K27me3, or DNMT1, which is a classical DNA methyltransferase that catalyzes and maintains DNA methylation, thereby epigenetically downregulating target gene expression (61). Many other lncRNAs, such as ANRIL (62), Xist (63), and HOTAIR (64), have been found to be capable of binding and recruiting EZH2 to exert their functions in the progression of HCC, indicating the close interaction between lncRNAs and EZH2-mediated hypermethylation. In addition, the demethylation of genes was also discovered in HCC progression. For example, lncRNA UPAT interacts with UHF1 and protects it from degradation, therefore inducing DNA hypomethylation to promote HCC progression (65, 66). All the evidence suggests that targeting lncRNA-induced epigenetic regulation might be a new treatment strategy for HCC.

#### Sponging miRNA

The competing endogenous RNA (ceRNA) mechanism is a crucial mechanism by which lncRNAs indirectly regulate gene expression (67, 68). LncRNAs can sponge miRNAs, which affects the expression of miRNA-targeted genes (69). A growing body of evidence shows that lncRNAs acting as ceRNAs play a significant role in regulating tumor cell proliferation, migration, invasion, apoptosis, immune evasion and drug treatment response (70–73). For instance, lncRNA LNC00667 plays an oncogenic role by binding to miR-130a-3p, thereby attenuating the inhibition of AR expression and promoting the HCC cell malignant phenotype (71). LncRNA PICSAR is upregulated in tissues, and gain- and loss-of-function experiments indicated that lncRNA PICSAR enhances cell proliferation and cell cycle progression, inhibits apoptosis by sponging miR-194-5p and subsequently upregulates eukaryotic initiation factor 6 (EIF6) expression in HCC (74). Moreover, several ceRNA networks of lncRNA-miRNA-target genes have been screened as prognostic models for HCC, but further *in vitro* and *in vivo* experiments to verify these networks are necessary in these studies.

#### Modulation of Transcription Regulators

In the nucleus, lncRNAs can modulate transcriptional regulators that have activating or repressive activities (75). For example, the tumor-suppressive lncRNA maternally expressed gene 3 (MEG3) interacts with the p53 DNA binding domain to promote p53-mediated transcriptional activity and induce the expression of various p53 target genes in HCC cells (76).

#### mRNA Stability

Generally, lncRNAs indirectly regulate the stability of mRNAs. For example, UFC1 promotes the stability of β-catenin mRNA by binding with the mRNA stabilizing protein HuR, increasing the levels of β-catenin and promoting HCC progression (77). lncRNA growth-arrested DNA damage-inducible gene 7 (gadd7) mediates mRNA decay by binding to TAR DNA-binding protein (78). The same research group found that lncRNAs directly bind with mRNAs to affect mRNA stability in the cytoplasm. For instance, lncRNAs transactivate STAU1 and mediate the decay of mRNA STAU1 by binding to the 3’-untranslated regions (3’ UTRs) of STAU1 (79).

### Functional Mechanisms of circRNAs in HCC

miRNAs can act as miRNA sponges, participate in the splicing of target genes, translate genes into proteins and interact with RNA binding proteins (RBPs) (80). The subcellular localization of circRNAs is essential for understanding the functional mechanism. Ample evidence has demonstrated that circRNAs are abundant in the cytoplasm and regulate miRNA function at the posttranscriptional level by sponging miRNAs. For example, circRNA 104718 promotes HCC cell proliferation, migration, invasion, and tumor growth and inhibits apoptosis through the regulation of thioredoxin domain-containing protein 5 (TXNDC5) by sponging miRNA 218-5p (81). However, a few circRNAs are distributed in the nucleus and contribute to modulating gene expression at the transcriptional and posttranscriptional levels (82–84). For example, circRNA ankrd52 is abundant in the nucleus and accumulates at transcription sites, interacting with Pol II elongation machinery and regulating local gene expression (85).

### Functional Mechanisms of microRNAs in HCC

miRNAs can exert both tumor-suppressive and oncogenic effects in HCC progression (86, 87). miRNAs regulate gene expression through degradation of their target mRNAs or suppression of translation by binding the 3’ UTRs of mRNAs. Each miRNA has the capacity to repress a large number of target genes; thus, mRNAs are strong regulators of gene expression (87). For example, miR-21 acts as a tumor promoter by targeting Kruppel-like factor 5, calmodulin-regulated spectrin-associated protein family member 1, DEAD-box helicase 1, MARCKS-like 1, etc (88–90).

## ncRNAs Regulate Cellular Components of the TME

Most studies have focused on the role of ncRNAs in the regulation of HCC cell functions and target genes. With advances in bioinformatics and next-generation sequencing, an increasing number of studies have shown that ncRNAs participate in cell-to-cell communication and regulate tumor immune cell activation, proliferation and cytokine secretion (91), thereby affecting tumor invasion, metastasis and immune system escape (92–95). Many miRNAs have been found to play important roles in the crosstalk between HCC cells and the TME (**Table 4**). Most lncRNAs and circRNAs exert their regulatory function in the TME of HCC by sponging the corresponding miRNAs (**Table 5**).

**Table 4:** The abnormally expressed miRNAs involved in functional regulation of immune cells.

miRNA	Expression	Detectable location	Target gene and pathway	Immune target	References
miR-570	Downregulated	Cell lines	/	CD3+CD4+T cells and CD3+CD8+IFN-γ+ T cells	(96)
miR-132	Upregulated	T helper 17 cells	SNIP1	Th17 cell differentiation and improves the function of Th17	(97)
miR-34a	Downregulated	Cells and tissues	CCL22 signaling	Induces Treg cell recruitment	(98)
miR-146a	/	/	STAT3	NK cell dysfunction	(99)
miR-889	Upregulated	Tissues	MICB	NK cell-mediated cytotoxicity	(100)
miR-615-5p	Upregulated	NK cells	IGF-1R (repression)	NK cells cytotoxicity	(101)
miR-223-3p	/	/	/	secretion of IL-1β and IL-18	(88)
miR-370	Downregulated	Cells and tissues	ISG15	Influences IFN-α sensitivity	(91)
miR-506	Downregulated	NK cells	STAT3	NK cell cytotoxicity	(102)
miR-26b-5p	Downregulated	Cells and tissues	PIM-2	Promotes cytokine secretion in CD4+ and CD8+ cells	(103)
miR-561-5p	Upregulated	Tissues and cell lines	CX3CL1	CX3CR1+ NK cells infiltration	(104)
miR-144/miR-451a	Downregulated	Cell lines and tissues	HGF	Macrophage M1 polarization and antitumor activity	(24)
miR-124	Downregulated	Liver fibrosis Tissues	IQGAP1 through the NF-κB pathway	LX-2 cells, TNF-α, IL-1β and IL-6	(89)
miR-15a/16-1	Downregulated	Tissues	NF-κB1	Disrupts the communication between Kupffer cells and Tregs	(105)
miR-152	Downregulated	/	/	NK and T cells	(106)

**Table 5:** The abnormally expressed lncRNAs and cirRNAs involved in functional regulation of immune cells.

ncRNA type	Name	Expression	Downstream molecules or signaling pathways	Immune target	Function in HCC cells	References
lncRNA	EGFR	Upregulated	/	Treg differentiation, suppresses CTL activity	Enhances tumor growth	(107)
lncRNA	cox-2	/	/	increasing the ability of M1 macrophages, inhibiting the polarization of M2 macrophages	Inhibits apoptosis, Enhances EMT and Metastasis	(108)
lncRNA	Tim3	Upregulated	Lck/NFAT1/AP-1 signaling, Tim-3–Bat3 signaling	Exacerbates Tim-3+ CD8 T cell exhaustion	Inhibits apoptosis	(109)
lncRNA	TUC339	Upregulated	/	Macrophage M1/M2 polarization	/	(110)
lncRNA	FENDRR	Downregulated	miR-423-5p/GADD45B	Suppresses Treg infiltration	Enhances proliferation and inhibits apoptosis	(111)
lncRNA	MALAT1	Upregulated	miR-140/VEGF-A	Facilitates the polarization of macrophage toward the M1 subset	Enhances metastasis and invasion	(112)
lncRNA	KCNQ1OT1	Upregulated	miR-506/PD-L1	T cell apoptosis	Enhances sorafenib resistance	(113)
lncRNA	PCED1B-AS1	Upregulated	miR-194-5p/PD-Ls	Immune suppression of T cells and macrophages	Enhances proliferation and inhibits apoptosis	(114)
lncRNA	NNT-AS1	Upregulated	TGF-β signaling pathway	CD4 T cell infiltration	/	(115)
lncRNA	XIST	Upregulated	miR-155-5p/PD-1/PD-L1.	/	/	(116)
lncRNA	lnc00638	/	miR-4732-3P/ULBP1/PD-L1	NK cell infiltration	/	(117)
circRNA	cir0110102	Downregulated	miR-580-5p/PPARα/CCL2 pathway	Macrophage activation	Inhibits proliferation, migration, and invasion	(118)
circRNA	MET	Upregulated	miR-30-5p/Snail/dipeptidyl peptidase 4(DPP4)/CXCL10	CD8+ T cell infiltration	Enhances invasion and metastasis	(119)
circRNA	cir0074854	Downregulated	/	Macrophage M2 Polarization	Inhibits migration and invasion	(120)
circRNA	cir0007456	Downregulated	miR-6852-3p/ICAM-1	Susceptibility to NK cells	/	(121)

### The Regulatory Role of miRNAs in TME of HCC

#### miRNAs and T Cells

T cells play an important role in the immune response against various pathogens and cancer cells (122). The T cell activation process is complex and involves many transcriptional and posttranscriptional regulators, including miRNAs (122). Lin et al. (96) reported that miR-570 was downregulated in HCC cell lines (including Bel-7404, Huh-7, and HepG2 cells), and the results of flow cytometry showed that the ratio of CD8+IFN-c+ T cells was markedly higher, while the ratio of CD3+CD4+ T cells was lower in the peripheral blood of nude mice injected with miR-570 mimics than that of those injected with NC-transfected SMMC7721 cells, suggesting that miR-570 might play a key role in the immune escape of HCC. Additionally, the expression of miR-26b-5 was lower in HCC tissues and cells than in normal controls and was associated with poor outcomes in HCC patients. Mechanistically, anti-miR-26b-5 mediates immunosuppression by regulating the secretion of tumor necrosis factor α (TNF-α), interferon-γ (IFN-γ), and interleukin-6 (IL-6) and IL-2 in CD4+ and CD8+ cells by targeting proviral integrations of moloney virus 2 (PIM2) in HCC (103).

Some studies have also explored the function of miRNAs in Th17 cells in the TME of HCC. During Th17 cell differentiation, treatment with the miR-132 mimic promoted the differentiation of Th17 cells, leading to a higher percentage of Th17+ cells and higher secretion of IL-17 and IL-22 (97). Furthermore, miR-132-overexpressing Th17 cells could enhance the activation of hepatic stellate cells (HSCs), inducing HCC cell EMT and migration (97).

Regulatory T cells (Tregs) normally play an immunosuppressive and tolerogenic role in the immune system and have been found to be coopted by tumor cells to escape immune surveillance (123). Yang et al. (98) reported that miR-34 exerts a tumor-suppressive function by affecting the TME by regulating the secretion of the chemokine CCL22, which facilitates the recruitment of Treg cells into the TME and immune suppression. Therefore, specific miRNAs might act as regulators of the TME during HCC progression.

#### miRNAs and Macrophages

TAMs play an essential role in the inflammatory microenvironment and mediate the initiation and progression of HCC according to their polarization state (M1/M2) (124). The miR-144/451a cluster was found to facilitate M1 macrophage polarization and antitumor activity in HCC (25). Mechanistically, silencing the CpG island of the miR-144/miR-451a promoter drove chromatin conformation remodeling, which promoted miR-144/miR-451a expression and further controlled TAM development and differentiated CD8+ cytotoxic T cells (25). Liu et al. (105) reported that hydrodynamic injection of miR-15a/16-1 inhibited the progression of HCC in mouse models, inhibited hepatic enrichment of Tregs and accelerated the recruitment of hepatic CD8+ cytotoxic T cells.

#### miRNAs and NK Cells

The expression of miR-615-5p is upregulated in NK cells of HCC patients compared with those of healthy controls. Silencing the expression of miR-615-5p impaired NK cytotoxicity and forced the expression of cytotoxic markers NKG2D, TNF-α and perforin (101). Bian et al. (106) found that downregulated miR-152 could cause epigenetic changes in HCC tumorigenesis, leading to the upregulation of human leucocyte antigen-G (HLA-G). HLA-G is a class of nonclassical MHC-I family members and is considered an immunosuppressive molecule that can bind to its receptor on NK and T cells, leading to an active immunosuppressive signaling pathway. In another study, the results of gain- and loss-of function assays in a mouse model showed that miR-561-5p was correlated with tumorigenesis and metastasis. Notably, upregulated miR-561-5p suppressed CX3CR1+ NK cell infiltration and function by targeting (C-X3-C motif) ligand 1 (CX3CL1) (104). Furthermore, miR-146a (99), miR-889 (100), and miR-506 (102) were found to regulate NK cell function and cytotoxicity against HCC cells, suggesting that modulating the expression of specific miRNAs could be a potential method to enhance NK cell-based antitumor treatments.

### The Regulatory Role of lncRNAs in TME of HCC

#### lncRNAs and T Cells

Increasing evidence has demonstrated that lncRNAs are involved in HCC progression and immune escape. For instance, lncRNA fetal-lethal noncoding developmental regulatory (FENDRR) acts as a miR-423-5p sponge to inhibit the immune-suppressive capacity of Tregs, therefore impairing the immune escape of HCC (111). Conversely, lncRNA epidermal growth factor receptor is highly expressed in Tregs and stimulates Treg differentiation, thus promoting HCC immune evasion and progression (107). Furthermore, lncRNA Tims and lncNNT-AS1 influence the outcome and immunotherapeutic response by decreasing tumor CD4 T cell and CD8 T cell infiltration, respectively (109, 115). Long noncoding RNA KCNQ1 overlapping transcript 1 (lncRNA KCNQ1OT1) has been shown to contribute to drug resistance and programmed death−ligand−1 (PD-L1)-mediated immune escape by sponging miR−506 in HCC cells (113). lncRNA X-inactive specific transcript (XIST) was also reported to regulate the expression of PD-1/PD-L1 by sharing a pathway between miR-194-5p and miR-155-5p in HCC (116).

#### lncRNAs and Macrophage Cells

Macrophages respond to environmental signals in two polarized ways: classical proinflammatory activation (M1) and alternative anti-inflammatory activation (M2). Many lncRNAs have been found to participate in the regulation of macrophage M1/M2 polarization, such as lnc TUC339 (110), MALAT1 (112), cox-2 (108) and PCED1B-AS1 (114). For example, it was observed in M1 macrophages that lncRNA cox-2 expression was increased compared with that in nonpolarized macrophages and M2 macrophages. Silencing the lncRNA cox-2 decreased the expression levels of proinflammatory factors in M1 macrophages while enhancing the expression levels of anti-inflammatory cytokines in M2 macrophages, suggesting that lncRNA cox-2 suppresses HCC immune evasion and tumor progression by regulating M1/M2 macrophage polarization (108).

#### lncRNA and NK Cells

An increasing number of studies have indicated the important role of lncRNAs in NK cell development and function (117, 125, 126). For instance, lncRNA GAS5 deficiency in activated NK cells has been demonstrated to reduce IFN-γ secretion, NK cell killing effects, and the apoptosis rate of HepG2 and Huh7 cells (126). However, the mechanism by which lncRNAs regulate NK cells is very complicated, and there are still relatively few related studies at present.

### The Regulatory Role of circRNA in TME of HCC

**Figure 2:**
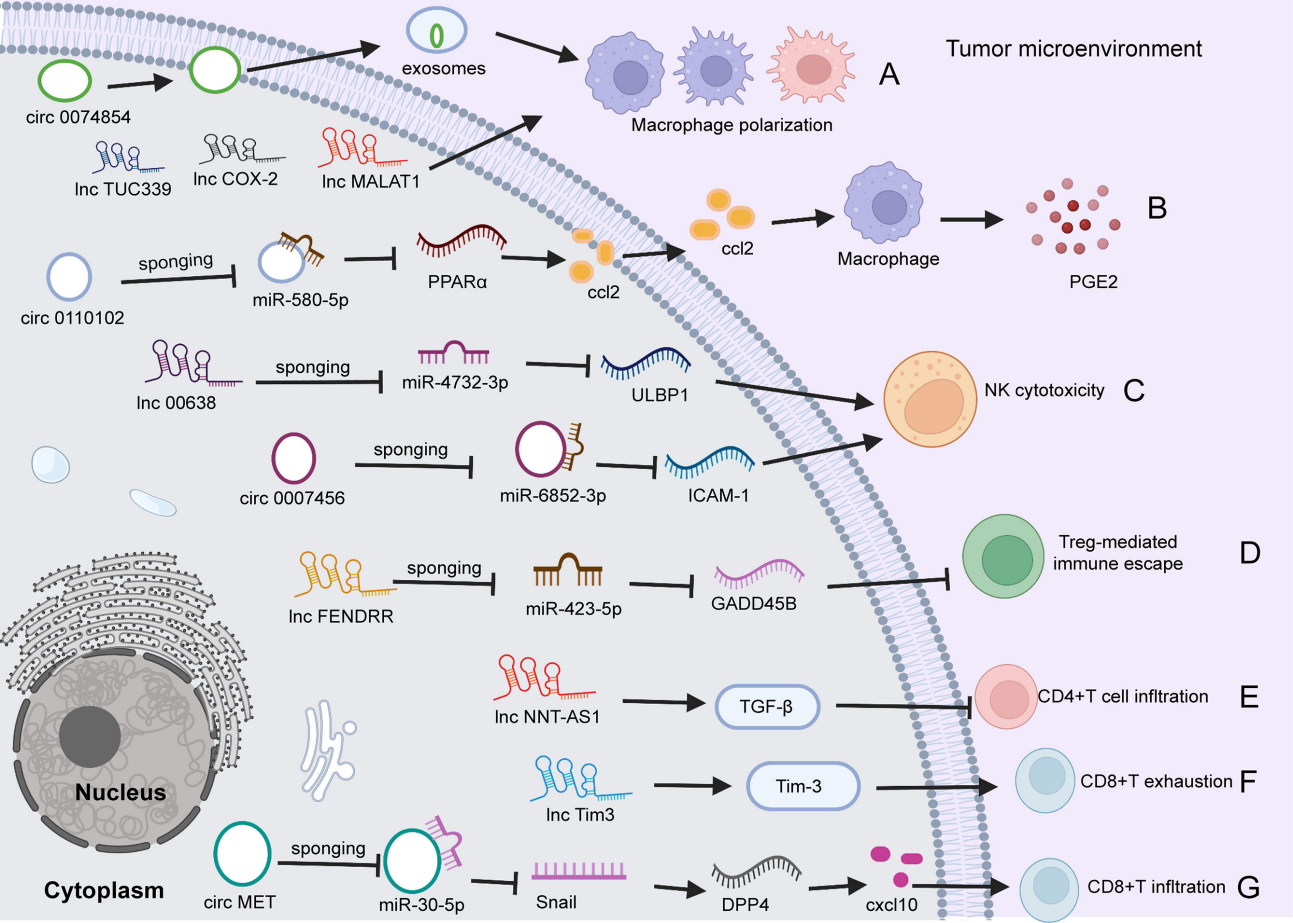
The role of circRNAs and lncRNAs in regulating HCC immunity. **(A)** circ0074854 in exosomes transfers from HCC cells to macrophages, regulating macrophage polarization. Besides, lncTUC339, lncCOX-2, and lncMALAT1 involve in macrophage polarization. **(B)** Upregulated circ0110102 promotes CCL2 secretion into the TME by reducing the level of PPARα by sponging miR-580-5P in HCC cells, leading to macrophage secretion of proinflammatory PGE2. **(C)** circ0007456 is downregulated in HCC cells and regulates NK cytotoxicity by mediating ICAM-1 expression by sponging miR-6852-3P. Similarly, lnc00638/miR-4732-3p/ULBP1 axis regulates NK cell cytotoxicity. **(D)** lncFENDRR acts as a miR-423-5p sponge to suppress the Treg cell mediated immune escape. **(E)**. lncNNT-AS1 activates TGF-β signaling to decrease tumor CD4+T cell infiltration. **(F)** Lnc-Tim3 exacerbates CD8 T cell exhaustion *via* binding to Tim-3. **(G)** circ MET is upregulated in HCC tumors, and overexpressed circMET facilitates CD8+T cells infiltration through the miR-30-5p/Snail/DPP4/CXCL10 axis. TME, tumor microenvironment; circRNA, circular RNA; PPARα, peroxisome proliferator-activated receptor α; miR, microRNA; DPP4, dipeptidyl peptidase 4; NK, natural killer; ICAM-1, intercellular adhesion molecule 1; PGE2, Prostaglandin E2; NK, natural killer; ICAM-1, intercellular adhesion molecule-1; ULBP1, UL16-binding protein 1; Treg, regulatory; TGF, Transforming growth factor; DPP4, dipeptidyl peptidase; CXCL10, CXC chemokine ligand 10.

Emerging evidence demonstrates that specific circRNAs play regulatory roles in innate and adaptive immune pathways (118, 119, 121). For example, circ0110102 was found to be upregulated in HCC tissues versus normal tissues, and this upregulation was correlated with a short survival time. Functionally, silencing circ0110102 enhanced tumor cell proliferation, migration, and invasion. Mechanistically, circ0110102 targeted miR-580-5p and inhibited CCL2 secretion into the TME by reducing the expression of peroxisome proliferator-activated receptor α (PPARα) in HCC cells and then suppressing macrophage secretion of proinflammatory cytokines by regulating the COX-2/PGE2 pathway (118). The expression of circRNA circ-0007456 is low in HCC tissues, and this miRNA functions by sponging miR-6852-3p to regulate the expression of intercellular adhesion molecule-1 (ICAM-1), resulting in a reduction in NK cytotoxicity toward HCC cells (127). Huang et al. (119) found that circ MET (circ 0082002) was upregulated in HCC tumors, and that circ MET overexpression facilitated HCC invasion, metastasis and immune suppression through the miR-30-5p/Snail/dipeptidyl peptidase 4 (DPP4)/CXCL10 axis. In addition, CXCL10 regulated CD8+ lymphocyte infiltration through the PD-1/PD-L1 pathway (119). In addition, exosomes are important messengers of intercellular communication (120, 128). Wang et al. (120) reported that hsa_circ_0074854 in exosomes could transfer from HCC cells to macrophages. Silencing hsa_circ_0074854 in exosomes inhibited macrophage M2 polarization, which impaired the migration and invasion of HCC cells (120) (**Figure 2**).

## Diagnostic, Prognostic and Therapeutic Potential of ncRNAs in HCC

HCC is one of the most common malignancies and ranks as the fourth leading cause of cancer-related death globally. The major reason is that most of the patients are diagnosed at an advanced stage. Therefore, exploration of diagnostic and predictive biomarkers for HCC patients in the early stage is needed. Specific ncRNAs released from cancer cells and tissues exist in exosomes, which are small membrane-derived vesicles. Interestingly, circulating exosomes can serve as vesicles loaded with ncRNAs (129–131). Previously, miRNAs have been identified in exosomes of biological fluids (including urine, serum and plasma) (132–134). More recently, various lncRNAs and circRNAs were applied as noninvasive diagnostic biomarkers for HCC due to their dysregulated expression in body fluids (135–137). Because obtaining biological fluids is noninvasive and can be repeated and because ncRNAs commonly have tissue specificity, ncRNAs in serum or exosomes are more ideal candidates for diagnostic biomarkers than those in tissues.

Furthermore, an increasing number of studies have confirmed that several ncRNAs might serve as promising prognostic biomarkers and therapeutic targets for the treatment of HCC. Numerous circRNAs, lncRNAs and miRNAs have been found to be dysregulated in tumor tissues, tumor cells, immune cells, plasma, and exosomes and to be markedly correlated with the prognosis of HCC patients, even with the activation of immune cell function involving the remodeling of TAMs. Therapeutically targeting tumor-promoting ncRNAs or restoring the function of ncRNAs with tumor-suppressive function might be promising methods. In fact, the regulatory networks of ncRNAs are extremely complicated because ncRNAs work with other biomolecules, such as coding RNAs, ncRNAs, DNAs and proteins (138). A better understanding of the regulatory mechanism of ncRNAs is necessary before ncRNAs can be tailored to therapeutic applications.

## Conclusion

Various ncRNAs have been found to be aberrantly expressed in HCC and are regulated by different mechanisms. Here, we mainly focused on the dysregulation of epigenetic mechanisms and genetic alterations, such as CNVs, histone modification, DNA methylation, and RNA methylation. Although ncRNAs do not encode proteins, they regulate gene expression levels at various levels (epigenetic regulation, transcriptional regulation and posttranscriptional regulation, etc.) in the form of RNA. Additionally, ncRNAs play important roles in the TME and are involved in the regulation of tumor cell proliferation, invasion, migration, immune cell infiltration and functional activation. Because ncRNAs serve as either tumor suppressors or oncogenes in the progression of HCC, a better understanding of the role and related regulatory mechanism is necessary for the development of promising prognostic/predictive biomarkers and potential targeted treatments.

## Author Contributions

LL and JL designed and guided the study. CX and XG wrote and edited the manuscript. ZB and YS helped with reference collection. All authors read and approved the final manuscript.

## Funding

This work was funded by the National Key Research and Development Program of China (2021YFC2301804, and 2021YFA1301104), and the National Nature Science Foundation of China (U20A20343).

## Conflict of Interest

The authors declare that the research was conducted in the absence of any commercial or financial relationships that could be construed as a potential conflict of interest.

## Publisher’s Note

All claims expressed in this article are solely those of the authors and do not necessarily represent those of their affiliated organizations, or those of the publisher, the editors and the reviewers. Any product that may be evaluated in this article, or claim that may be made by its manufacturer, is not guaranteed or endorsed by the publisher.
